# Learning from Nepal’s Progress to Inform the Path to the Sustainable Development Goals for Health, Leaving No-One Behind

**DOI:** 10.1007/s10995-020-02899-2

**Published:** 2020-02-21

**Authors:** Joy E. Lawn, K. C. Ashish

**Affiliations:** 1 MARCH Centre, London School of Hygiene & Tropical Medicine, London, UK; 2 Department of Women’s and Children’s Health, Uppsala University, Uppsala, Sweden

The health of Nepal’s women and children has changed in many ways since 1990, the baseline of the Millennium Development Goals (MDGs). Under-five mortality rate has decreased by more than 75%, and maternal mortality ratio by more than 70% (UNICEF 2019; WHO 2019). Nepal was one of very few low-income countries to meet both MDG4 (child survival) and MDG5 (maternal health). This success story has been applauded, and whilst it is clear that many factors contributed, Nepal’s consistent focus on primary health care, plus early adoption of innovations, likely played a significant role (Moucheraud et al. 2016; Pradhan et al. 2012).

In the Sustainable Development Goal era, only one of the 17 SDGs (SDG3) is on health, and within SDG3 women’s and children’s health are alongside other emerging health priorities. Yet there are still over 8 million deaths per year, including stillbirths, neonatal, child and maternal deaths (UNICEF 2019). Progress for maternal, neonatal and child mortality in the SDG era, let alone acceleration, requires ongoing attention to implementation, but also new ideas.

In this special series on maternal, neonatal, child health and nutrition progress in Nepal, the co-authors dig into not only the progress made but also what needs to be done differently for health, including linking to other SDG themes such as climate, and gender equality. Nepal’s national Demographic Health Surveys and Nepal Health Facility Survey were the main inputs for analysing progress and making projections to 2030. The seven papers (Table 1) focus on important principles of (1) ending preventable deaths with equity; (2) providing Universal Health Coverage (UHC), with high quality care, especially at birth; (3) going beyond survival and (4) reaching beyond health through multi-sectoral collaboration (Budhathoki et al. 2019a, b; Gurung et al. 2019; Kc et al. 2019a, b, c; Sunny et al. 2019; Thapa et al. 2019).

**Fig. 1:**
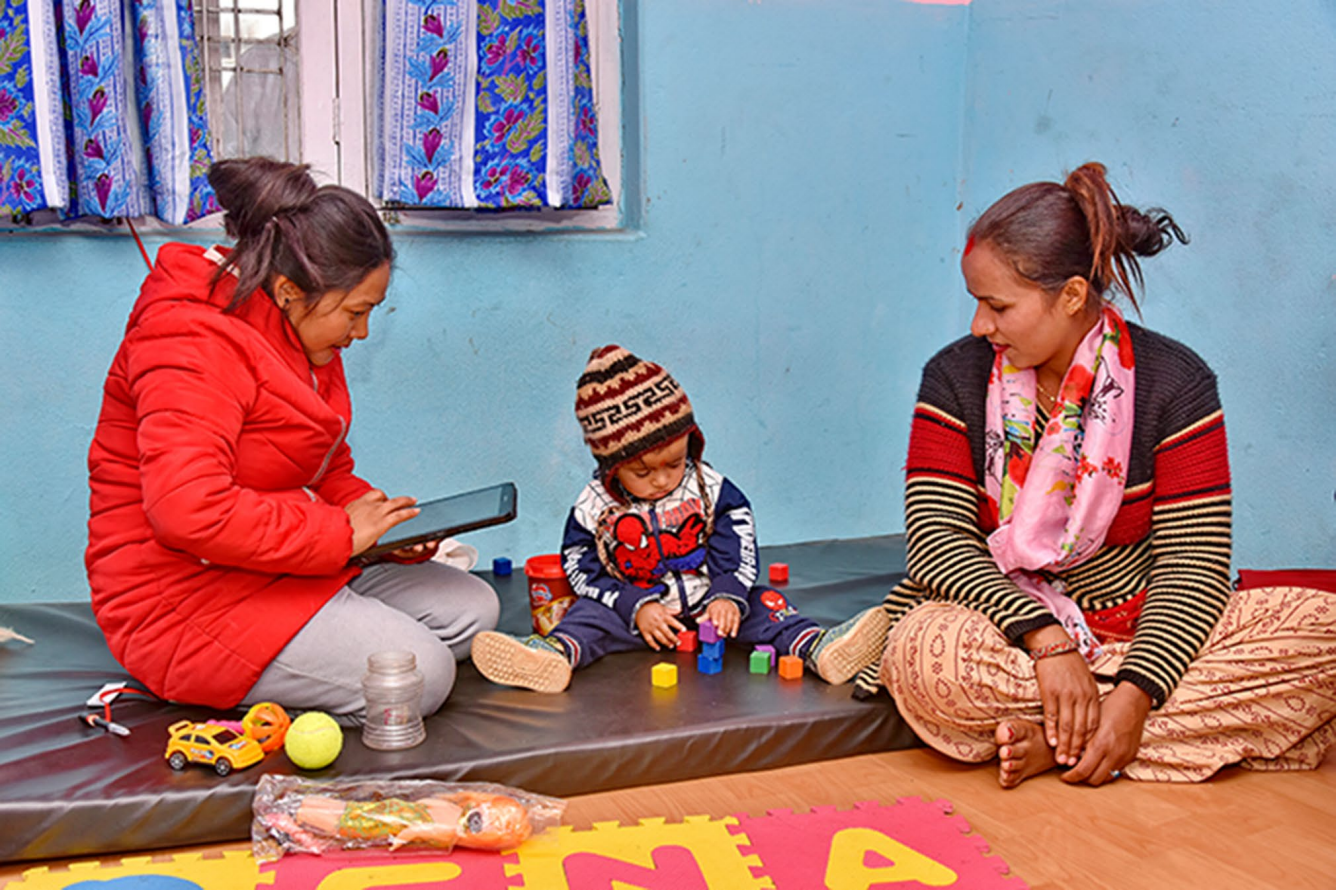
Child in the EN-SMILING study with their mother and a primary care assessor trained in child development

1. *Ending preventable deaths with equity* Nepal’s remarkable progress in reducing under-five deaths has not yet been achieved for neonatal deaths, and the progress has been unequal for the poorest families compared to the wealthiest. Newborn deaths act as an indicator for the most vulnerable. If Nepal’s current trends continue, the poorest group will not attain the 2030 SDG for newborns until 40 years too late (Kc et al. 2019a, b, c). Increased focus on these poorest families is required for the health and survival of women and their newborns (Thapa et al. 2019). Inequalities for antenatal care and skilled birth attendance have widened. In addition, stillbirths, which were left out of the MDGs, account for 2.6 million deaths worldwide, and are a major stigma for women (Lawn et al. 2016). Nepal is one of the few countries with a stillbirth target for 2030, but also needs focused strategies to meet this target (Gurung et al. 2019).
2. *Providing UHC with quality of care especially around the time of birth* Care at birth, and especially for vulnerable small and sick newborns, is a sensitive marker of UHC, as underlined in the Every Newborn Action Plan and also Nepal’s national plan (Ministry of Health and Population 2016). Yet these new analyses show that many facilities do not have the basic infrastructure, equipment and drugs for high quality care (Kc et al. 2019a, b, c). Nepal is leading innovations in this area, with large studies to improve this quality of care (Kc et al. 2019a, b, c), and also the multi-country EN-BIRTH study to validate measures for coverage and quality of care in routine systems (Day et al. 2019).
3. *Going beyond survival* An important shift in the post-MDG era is to go beyond survival alone, for children (child development, optimal nutrition), for women (preventing morbidly, addressing maternal mental health) and for adolescents (transition to healthy behaviours, optimal education). Nutritional outcomes in children often require intergenerational improvement for girls and women (Budhathoki et al. 2019a, b). Optimal child development is strongly associated with nutritional status and can be two-way, since children with disability are especially at risk of failing to thrive. Nepal is also innovating in these areas, being part of the Every Newborn—Simplified Measurement Integrating Longitudinal Neurodevelopment & Growth (EN-SMILING) multi-country study following up children at risk of developmental delay and measuring both child development outcomes and anthropometry (Fig. 1).
4. *Reaching beyond health through multi-sectoral collaboration* Gender equity and education of girls and women is foundational for women’s health and wellbeing, and also that of their children (Gurung et al. 2019). Improving gender equality is associated with education but also access to finances (Sunny et al. 2019). Indoor pollution has been associated with pneumonia and addressing pollution is another factor in improving health outcomes, for example, prevention of pneumonia in children (Budhathoki et al. 2019a, b).

**Table 1:** Series of papers on Nepal’s progress in maternal, newborn, child health and nutrition

Paper number	Paper title
1	Trends for neonatal deaths in Nepal (2001–2016) to project progress towards the SDG target in 2030, and risk factor analyses to focus action (Kc et al. 2019a, b, c)
2	The association of women’s empowerment with stillbirths in Nepal (Gurung et al. 2019)
3	Equity and coverage in the continuum of reproductive, maternal, newborn and child health services in Nepal: projecting the estimates on death averted using the LiST Tool (Thapa et al. 2019)
4	Quality of care for maternal and newborn health in health facilities in Nepal (Kc et al. 2019a, b, c)
5	Stunting among under 5-year-olds in Nepal—trends and risk factors (Budhathoki et al. 2019a, b)
6	The association of childhood pneumonia with household air pollution in Nepal: evidence from Nepal Demographic Health Surveys (Budhathoki et al. 2019a, b)
7	Out of Pocket Expenditure for sick newborn care in referral hospitals of Nepal (Sunny et al. 2019)

Nepal has shifted to a decentralized system, providing an opportunity for evidence-based planning in each local context. Yet this shift is also a time of risk and requires many more leaders and implementers at all levels. The newly decentralized system requires adequate administrative capacity at the local and community level to identify the women’s and children’s issue, allocate resource and implement multi-sectoral interventions. Empowered leadership at the local level to set up infra-structure and systems to improve quality of care for women and children will reduce the inequity gap in the long run.

I hope that this special series will help guide programme design for quality and equity in Nepal and beyond. With just 10 years remaining to meet the SDGs, these papers give insights, and also warnings, for the path ahead. Using the learnings from Nepal, to further reduce maternal, neonatal and child mortality, other countries with similar settings need to increase investment for interventions which reduces inequity gap and improve quality of care. Now is the time to accelerate, to innovate and especially, to institute intentional pro-poor approaches to make sure Nepal will also meet the health SDGs for women, newborns and children.
